# Reactivation of Latent Tuberculosis in Cynomolgus Macaques Infected with SIV Is Associated with Early Peripheral T Cell Depletion and Not Virus Load

**DOI:** 10.1371/journal.pone.0009611

**Published:** 2010-03-10

**Authors:** Collin R. Diedrich, Joshua T. Mattila, Edwin Klein, Chris Janssen, Jiayao Phuah, Timothy J. Sturgeon, Ronald C. Montelaro, Philana Ling Lin, JoAnne L. Flynn

**Affiliations:** 1 Department of Microbiology and Molecular Genetics, University of Pittsburgh School of Medicine, Pittsburgh, Pennsylvania, United States of America; 2 Division of Laboratory Animal Resources, University of Pittsburgh School of Medicine, Pittsburgh, Pennsylvania, United States of America; 3 Center for Vaccine Research, University of Pittsburgh School of Medicine, Pittsburgh, Pennsylvania, United States of America; 4 Department of Pediatrics, Children's Hospital of Pittsburgh of the University of Pittsburgh Medical Center, Pittsburgh, Pennsylvania, United States of America; Institute of Infectious Diseases and Molecular Medicine, South Africa

## Abstract

HIV-infected individuals with latent *Mycobacterium tuberculosis* (Mtb) infection are at significantly greater risk of reactivation tuberculosis (TB) than HIV-negative individuals with latent TB, even while CD4 T cell numbers are well preserved. Factors underlying high rates of reactivation are poorly understood and investigative tools are limited. We used cynomolgus macaques with latent TB co-infected with SIVmac251 to develop the first animal model of reactivated TB in HIV-infected humans to better explore these factors. All latent animals developed reactivated TB following SIV infection, with a variable time to reactivation (up to 11 months post-SIV). Reactivation was independent of virus load but correlated with depletion of peripheral T cells during acute SIV infection. Animals experiencing reactivation early after SIV infection (<17 weeks) had fewer CD4 T cells in the periphery and airways than animals reactivating in later phases of SIV infection. Co-infected animals had fewer T cells in involved lungs than SIV-negative animals with active TB despite similar T cell numbers in draining lymph nodes. Granulomas from these animals demonstrated histopathologic characteristics consistent with a chronically active disease process. These results suggest initial T cell depletion may strongly influence outcomes of HIV-Mtb co-infection.

## Introduction

Approximately 90% of human *Mycobacterium tuberculosis* (Mtb) infections are clinically latent and likely represent an immune response that successfully limits bacterial growth, resulting in persistence within multi-cellular structures called granulomas [1]. While granulomas are composed of many different cell types, macrophages and T cells are important components that collaborate to limit bacterial replication and prevent dissemination. The immune response of immunocompetent individuals can prevent active tuberculosis for years or decades, and latently infected individuals have only a 5–10% lifetime risk of developing reactivated tuberculosis (TB) [2]. Immunosuppressed individuals have a significantly greater chance of developing active disease, and TB is the leading killer of individuals infected with human immunodeficiency virus (HIV) [3]. In contrast to most opportunistic infections, which present in the later stages of HIV infection, TB afflicts HIV-positive individuals throughout the course of infection, even while CD4 numbers are well preserved [4], [5], [6]. While factors explaining the high rates of reactivated TB in co-infected humans remain unclear, depletion of CD4 T cells [7] and increased virus loads [8] within granulomatous tissue may be contributors.

Co-infections in humans, and the accompanying immune responses, are inherently difficult to investigate and studies are frequently confounded by uncontrolled variables. Our current understanding of immune responses to HIV-Mtb co-infection comes predominantly from human clinical studies [8], [9]. As with all clinical studies involving human subjects, there are limitations to studies that can be performed with HIV-Mtb co-infected individuals. Some challenges include difficulty determining which infection occurred first and when, limited availability of pre- and post-infection samples, restrictions on unnecessary invasive procedures to obtain tissue samples, and limited availability of post mortem tissue samples for immunologic analysis. Additionally, most HIV-TB clinical studies are in individuals with active tuberculosis, and cannot fully explore the events that precede or occur during reactivation. Human studies also have numerous uncontrolled variables including the undefined status of the immune system prior to infection and the presence of other undiagnosed co-infecting pathogens that may have an effect on the host immune response. Consequently, a biologically relevant animal model of HIV-Mtb co-infection where the amount and sites of sampling could be increased and the confounding variables minimized would be an extremely valuable asset.

Good animal models for HIV and TB exist, but there is not a model which recapitulates HIV-infection in an individual with latent TB. Macaques are frequently used to model HIV by infection with simian immunodeficiency virus (SIV) or SHIV, a HIV-SIV chimera. Depending on the macaque species and the virus type used, these animals can be excellent models for human infection and disease [10], [11], [12], [13], [14], [15], [16], [17]. Macaques are also valuable in studying tuberculosis [18], [19], [20], [21], [22], [23]. Cynomolgus macaques infected with a low number of Mtb bacilli develop clinical signs and pathology similar to humans with active TB or develop subclinical latent infections, with equal proportions of each infection outcome observed [18], [21]. Moreover, latency can be maintained for significant time periods. In our experience working with cynomolgus macaques over the past decade, only two of approximately 85 latently infected monkeys spontaneously reactivated [13,19, unpublished data]. Thus, cynomolgus macaques with latent TB have a <5% chance of spontaneously reactivating TB within a few years of infection and can maintain this latent state for years [18], [24].

Nonhuman primates have been used to examine interactions between SIV and mycobacteria. Macaques co-infected with SIV and Mtb (strain H37Rv) [25] or SIV and *M. bovis* BCG [20], [26] have been used to examine how mycobacteria induce AIDS-like symptoms. Rhesus (*M. mulatta*) or pigtail (*M. nemestrina*) macaques were inoculated with mycobacteria after SIV infection or simultaneously co-infected with BCG and SIV [20], [26]. These studies indicated that SIV could be immunosuppressive and sometimes exacerbate mycobacterial disease. Despite the range of model systems available, similar studies into disease processes underlying reactivated TB in HIV-infected humans with latent TB have yet to be done.

In the current study we use cynomolgus macaques infected with SIVmac251, a virulent HIV-like virus, to develop a novel model of HIV-induced reactivated TB. We used this model to examine immunologic, microbiologic and virologic changes in the peripheral blood and tissues that have not been extensively investigated in human or nonhuman primate HIV/SIV-Mtb co-infection studies. Co-infected animals showed a spectrum of disease severity, with the animals that reactivated <17 weeks post-SIV infection experiencing more severe pathology than animals that reactivated >26 weeks post-SIV infection. We found that time to reactivation correlated with significant peripheral CD4 T cell depletion but not virus load during the acute phase of SIV infection. The co-infected animals had fewer T cells in lung tissue than SIV-negative macaques with active TB, and a trend towards fewer T cells in the pulmonary lymph nodes than SIV-only macaques. We also present evidence of rapid impaired local control of latent Mtb infection, following SIV infection. These studies pave the way for detailed investigations of factors underlying reactivated TB in HIV-Mtb co-infected humans that were not previously possible.

## Materials and Methods

### Ethics Statement

All experimental manipulations and protocols were approved by the University of Pittsburgh School of Medicine Institutional Animal Care and Use Committee. The animals were housed and maintained in accordance with standards established in the Animal Welfare Act and the Guide for the Care and Use of Laboratory Animals.

### Experimental Animals

Fifteen adult (>4 years of age) cynomolgus macaques (*Macaca fascicularis*) of Chinese origin were used for these studies (Covance, Alice, TX; USA Valley Biosystems, West Sacramento, CA). Prior to the study, animals were tested to ensure they were free of *Mtb*, SIV, SHIV or simian retrovirus D infection. Animals were also given physical and clinical examinations including differential blood cell counts, erythrocyte sedimentation rate (ESR), serum chemistry profile and thoracic radiography to ensure they were free of underlying disease processes. All animals were given 14-day prophylactic Bactrim (sulfamethoxazole and trimethoprim) treatments to eliminate potential *Pneumocystis* colonization infection. SIV-infected animals were housed under BSL-2 conditions while *Mtb*-infected animals were housed under BSL-3 conditions. Bronchoalveolar lavage (BAL), gastric aspirate and other procedures were performed as previously described [18], [21]. We used historical controls from previous studies [18,21,unpublished data] on animals with active and latent TB to minimize the number of animals used in this study.

### Mtb and SIV Infection

The experimental design of these studies is described in figure S1. Ten animals were infected with ∼25 CFU Erdman strain *Mtb* via intra-bronchial instillation as previously described [21]. Mtb infection was confirmed in all ten animals by conversion of negative to positive tuberculin skin test and peripheral blood mononuclear cells (PBMC) responses elevated from baseline in lymphocyte proliferation (LPA) and PBMC enzyme-linked immunosorbent spot (ELISPOT) assays. Animals were classified as latent or active 8–10 months post infection with the criteria for latency defined as TST positive but with no signs of clinical disease as previously described [18], [21]. Of the original ten animals infected with Mtb, six were classified as latent and selected for SIV infection. A seventh latent monkey (10405) and infected with the same dose and strain of Mtb from another study, was added to this group to increase the number of animals.

For SIV infection, concentrated SIVmac251 stock (kindly provided by Dr. Keith Reimann, Beth Israel Deaconess Medical Center, Harvard University) was diluted in RPMI medium, and 10^6^–10^7^ TCID50 units of virus injected intravenously. Seven animals with latent TB and four Mtb-negative animals were infected with SIV, with the four Mtb-negative animals designated as the SIV-only control group. Historical data from latent and active control monkeys were used for comparison to minimize the number of animals needed for this study. Some data from these monkeys, as well as full characterization of pathology and disease outcome, has been published previously [18]. Criteria for assessing reactivation included prolonged weight or appetite loss, elevated erythrocyte sedimentation rate (ESR), Mtb-positive gastric aspirate or BAL cultures, or radiographic evidence of lung involvement, as shown in Table 1.

**Table 1:** Clinical evidence for reactivated TB in co-infected monkeys.

Monkey	Positive clinical indicator of TB (week observed)^a^	Necropsy^a^
2407	GA^b^ (4,9), increasing ESR (4,9,12), chest x-ray (9, 12)	12
1407	increasing ESR^b^ (4,9,13), chest x-ray (14)	15
1207	GA (10), chest x-ray (6)	17
1807	BAL^b^ (16,17)	26
1907	BAL (17,34), GA (37,44), chest x-ray (37)	45
3007	increasing ESR (41,45), progressive weight loss (37,41,45)	47
10405	chest x-ray (47)	48

a indicates weeks post-SIV infection.

b GA–gastric aspirate, ESR–erythrocyte sedimentation rate, BAL–bronchoalveolar lavage.

### Necropsy Procedures

Animals were humanely euthanized and necropsied as previously described [21] when indicators of active TB were present (in co-infected monkeys) or at the end of the study (11 months post-SIV). Necropsies were conducted by veterinarians with substantial experience examining Mtb-infected cynomolgus macaques (E.K., C.J.). Lung lobes, liver, spleen, and kidney were examined for evidence of tuberculous disease. Similarly, thoracic, axillary and inguinal lymph nodes were examined for the presence of granulomas. All gross pathology associated with tuberculous disease was recorded and quantified using a previously established scoring system validated to differentiate latent from active tuberculosis in this model [18]. Tissue (granulomas, lymph nodes, uninvolved lung from each lobe, extrapulmonary organs) was divided into pieces for histology and RNA isolation, and the remainder was mechanically homogenized into single-cell suspension for immunologic and microbiologic analysis and virus load determination. 30–40 samples per monkey were examined histologically and used for bacterial, virologic and immunologic assays. In several monkeys, gut tissue was obtained for analysis of T cell frequencies. Bacterial numbers in tissues were quantified by plating dilutions of tissue homogenate onto 7H10 agar plates and the number of colony forming units (CFU) in the original sample calculated after 4–6 weeks of incubation at 37°C/5% CO_2_.

### Immunologic Analysis

Blood was drawn from SIV-only and co-infected animals every week prior to and for the first eight weeks post infection. Thereafter, blood was drawn from Mtb-SIV co-infected animals every other week and SIV-only animals monthly. PBMC were isolated via percoll gradient centrifugation as previously described [27]. Axillary or inguinal lymph nodes were biopsied at pre-infection, 4, 8 and 16 weeks post SIV infection. PBMC and lymph node cells were subjected to flow cytometry and IFNγ ELISPOT assays. ELISPOT assays using tissue or BAL cells included autologous monocyte-derived dendritic cells, as antigen-presenting cells, as previously described [21], [27].

### ELISPOT Assays for Mtb- and SIV-Induced IFNγ Responses

ELISPOT assays were performed as previously described [21], [27] with 150,000 PBMC or lymph node cells per well using ELISPOT reagents with known cross reactivity against macaque IFNγ (MabTech, Mariemont, OH) [18]. *Mtb* antigens used in ELISPOT were peptide pools (overlapping 20-mers; 10 ug/mL) from CFP-10 (Rv3872) and ESAT-6 (Rv3875) synthesized by Sigma-Genosys (Woodlands, TX). Peptide pools for viral antigens gag, pol, env, tat, nef and rev (overlapping 20-mers) all used at 10 ug/mL, based on SIVmac239) were obtained from the AIDS Research and Reference Reagent Program (National Institutes of Health, Germantown, MD). Phorbol 12,13-dibutyrate (PDBu) and ionomycin (50 nM and 10 uM final concentration respectively; Sigma) were used as positive controls. Media-only wells were used as negative controls. Cells in ELISPOT assays were incubated with antigens for two days at 37°C/5% CO_2_ prior to being developed as previously described [27] and read using an ELISPOT plate reader (Cellular Technology LTD, Cleveland, OH). All conditions were performed in duplicate wells. ELISPOT data were normalized and expressed as SFU per 10^6^ cells.

### Flow Cytometry

All antibodies used for flow cytometry were direct conjugates against human proteins and obtained from BD Biosciences (San Jose, CA) unless otherwise noted. Approximately 1×10^6^ PBMC or tissue cells were stained using combinations of the following antibodies: CD3 (clone SP34-2), CD4 (clone L200), CD8 (clone DK25 [Dako; Carpinteria, CA], clone SK-1, clone OKT8 [eBioscience; San Diego, CA]) CD29 (clone HUTS-29) and CD69 (clone FN50) (clone 2H7 [eBioscience]). Cell phenotypes were read with a LSR II flow cytometer (BD Biosciences) and positively-stained populations gated using fluorochrome-matched isotype antibodies as negative controls using the FlowJo software package (Tree Star Inc., Ashland, OR).

### Virus Load Determination

Quantitative real time reverse transcriptase (qRT-PCR) for the SIV *gag* gene was used to measure virus loads as previously described [27], [28]. RNA was isolated from 1×10^6^ percoll gradient-isolated PBMC or lymph node cells, and from 2×10^5^ BAL or tissue cells using the Qiagen RNeasy kit (Valencia, CA). Plasma virus load was determined by isolating RNA from 200 uL of plasma using the Purelink Viral RNA/DNA mini kit (Invitrogen, Carlsbad, CA) as per manufacturer's instructions.

### Statistical Analysis

Data were analyzed using Prism (Graphpad Software, San Diego, CA). Pair-wise comparisons between groups (e.g. latent and co-infected animals) was performed using the Mann-Whitney test with p<0.05 considered statistically significant.

## Results

### CD4 T Cells Were Transiently Depleted in the Periphery of SIV-Infected Animals Coincident with Peak Virus Load in PBMC

**Figure 1:**
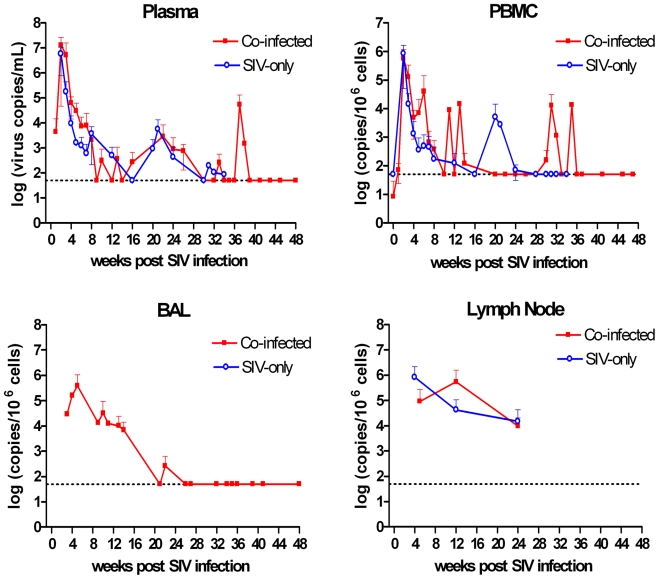
SIV loads in plasma and PBMC of Mtb-SIV co-infected and SIV-only monkeys. No significant differences in plasma, peripheral blood mononuclear cells (PBMC), bronchial alveolar lavage cells (BAL) or peripheral lymph node viral titer were observed between co-infected cynomolgus macaques (red line, n = 7) or the SIV-only group (blue line, n = 4). We were unable to obtain enough BAL cells from SIV-only monkeys to reliably measure virus load. The horizontal dashed line represents the detection limit for viral copy numbers. Lower error bars have been removed for clarity.

We measured viral titers in SIV-infected animals to determine how SIV infection correlated with reactivation or whether Mtb infection influenced SIV loads. SIV titers in plasma, PBMC, and lymph node cells were similar between co-infected monkeys and SIV-only monkeys (Figure 1). Peak virus loads in PBMC and plasma occurred at 2–3 weeks post SIV infection and then declined to low levels for the duration of the study. The greatest virus burden in BAL cells from co-infected monkeys occurred two weeks after peak virus load in the peripheral blood. Virus loads in cells from peripheral lymph nodes were not significantly different between time points, but these sites maintained moderate virus loads (10^4^–10^6^ copies/10^6^ cells). We also compared peripheral virus loads between co-infected and SIV-only monkeys to determine whether Mtb infection modified peripheral virus loads but did not find significant differences between these groups (data not shown).

### SIV Causes Reactivation of Latent Tuberculosis

We previously described the clinical, microbiologic, radiologic and immunologic parameters used to define latent infection and active disease in macaques and demonstrated that these are validated by quantitative measurements of bacterial burden and gross pathology at necropsy [18], [21]. Briefly, latent monkeys are asymptomatic and lack radiographic or microbiologic evidence of Mtb infection beyond three months post infection [18], [21]. Reactivation is defined as evidence of active TB either by microbiologic, radiographic or clinical parameters after a period of stable latent infection [18]. Based on these criteria, three of the seven co-infected monkeys with latent TB had findings consistent with reactivated TB 12–17 weeks post-SIV infection while the remaining four monkeys reactivated between 26–48 weeks post-SIV (Table 1). Monkey 1807 was necropsied after positive BALs and had relatively low necropsy and bacterial number scores, suggesting that positive cultures from BALs do not necessarily correlate with significant disease burden, but may signify the first sign of reactivation [18]. Considering this, Monkey 1907 was necropsied approximately 28 weeks after the first positive BAL and with additional positive clinical results to confirm reactivation was underway. While a spectrum of disease was observed, we loosely categorized the monkeys according to time of reactivation: early reactivation (reactivated TB in <17 weeks post SIV infection) and later reactivation (reactivated >26 weeks post SIV).

### Relationship between Peripheral T Cell Depletion and Virus Loads and Time to Reactivation

**Figure 2:**
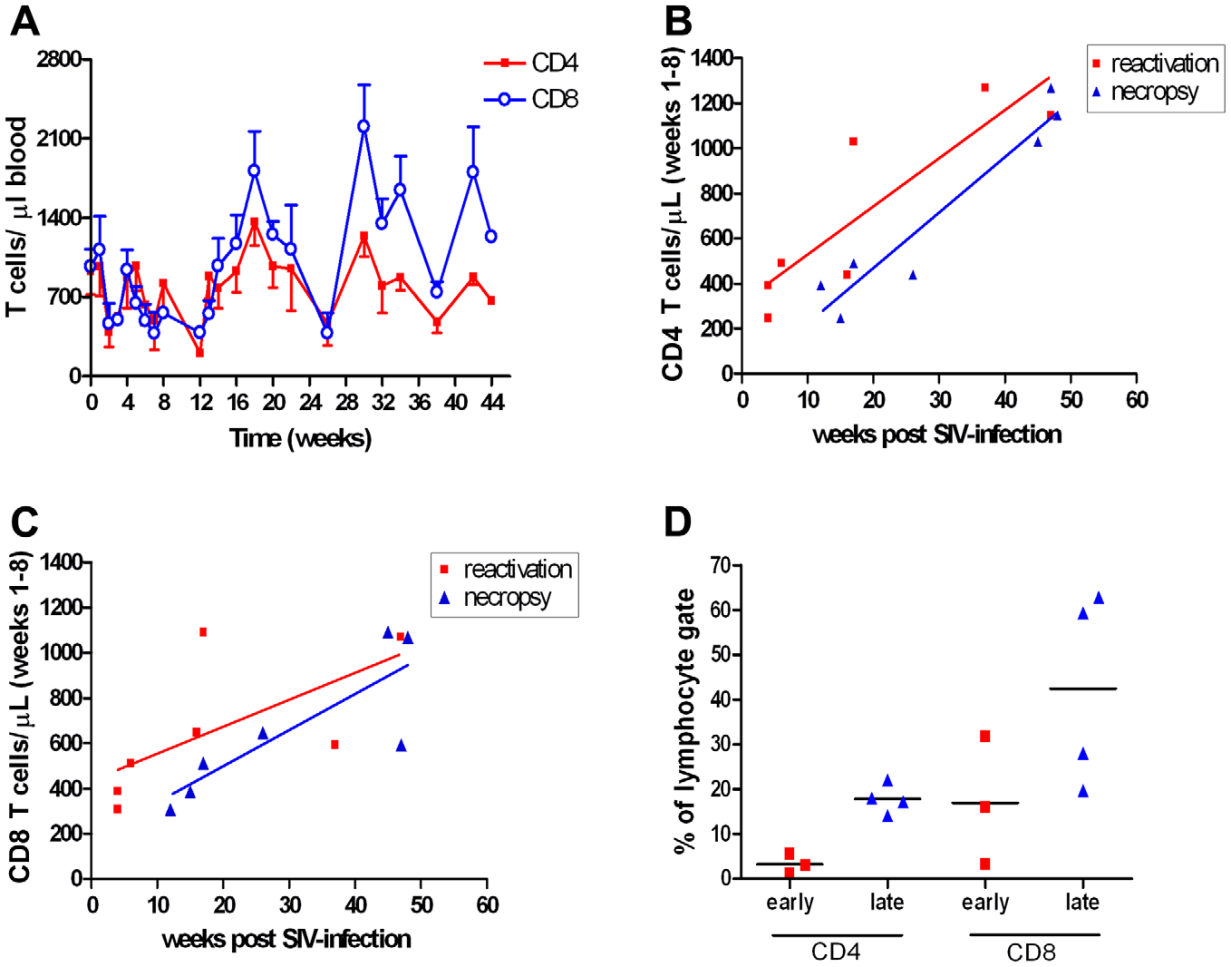
Acute CD4 T cell depletion correlates with reactivated TB. A. Mean CD4 and CD8 T cell numbers from whole blood of co-infected macaques. Upper (CD8) and lower (CD4) error bars have been omitted for clarity. B. Mean CD4 T cell numbers over weeks 1–8 correlate with reactivation (red line: P = 0.011, R2 = 0.756) and necropsy time (blue line: P = 0.0007, R2 = 0.9172). C. Mean CD8 T cell numbers do not correlate with reactivation (red line: P = 0.112, R2 = 0.426) but do correlate to necropsy time (blue line: P = 0.020, R2 = 0.692). D. Animals that reactivate <17 weeks post-SIV infection have a trend toward lower frequencies of CD4 T cell in the BAL at 10 weeks post SIV infection than monkeys >26 weeks post SIV infection Man-Whitney (P = 0.057). 1207 (early) BAL cells were sampled 8 weeks post SIV infection.

SIV infection affects lymphocyte numbers, and changes in CD4 and CD8 T cell numbers were followed in the PBMC and axillary or inguinal lymph nodes (referred to here as peripheral lymph nodes) of co-infected monkeys over the course of SIV infection to better understand the dynamics between lymphocyte numbers and reactivation. All monkeys experienced substantial declines in numbers of CD4 and CD8 T cells in the PBMC (Figure 2) and peripheral lymph nodes (Figure S2) between 2–4 weeks post SIV infection, a time corresponding with peak viremia (Figure 2A). After 8 weeks, T cells recovered to pre-SIV levels in all monkeys. No differences in CD4 or CD8 frequencies were found between SIV-only and co-infected animals (data not shown). Peripheral T cell numbers in co-infected animals were averaged during acute SIV infection (weeks 1–8 post-SIV) and plotted against the week that either indicators of reactivated TB were noted or the week that necropsy occurred to address the relationship between T cells and reactivation. Necropsy time was based on the aggregate clinical signs in a monkey. There was a significant correlation between extent of depletion of CD4 T cell numbers 2–8 weeks post-SIV and time to reactivation (P = 0.011, R^2^ = 0.755) and necropsy time (P = 0.0007, R^2^ = 0.9172) (Figure 2B). The correlation between reactivation signs and early depletion of CD8 T cell was not significant (P = 0.112, R^2^ = 0.425) but extent of early depletion of CD8 T cells did correlate with time of necropsy (P = 0.0202, R^2^ = 0.6923; Figure 2C). Although depletion in the periphery is an indicator of the overall effect of SIV infection, T cell depletion in the local granuloma environment is more likely to have an influence on reactivation. It was not possible to directly sample granulomas to measure T cell frequencies over the course of SIV infection (except at necropsy, see below), so we sampled the airways and analyzed BAL cells instead. The early-reactivating monkeys had lower frequencies of CD4 T cells (p = 0.0571) in the airway at 10 weeks post-SIV than monkeys that reactivated later (Figure 2D).

### Peripheral IFNγ Responses Increase in Response to SIV Infection

**Figure 3:**
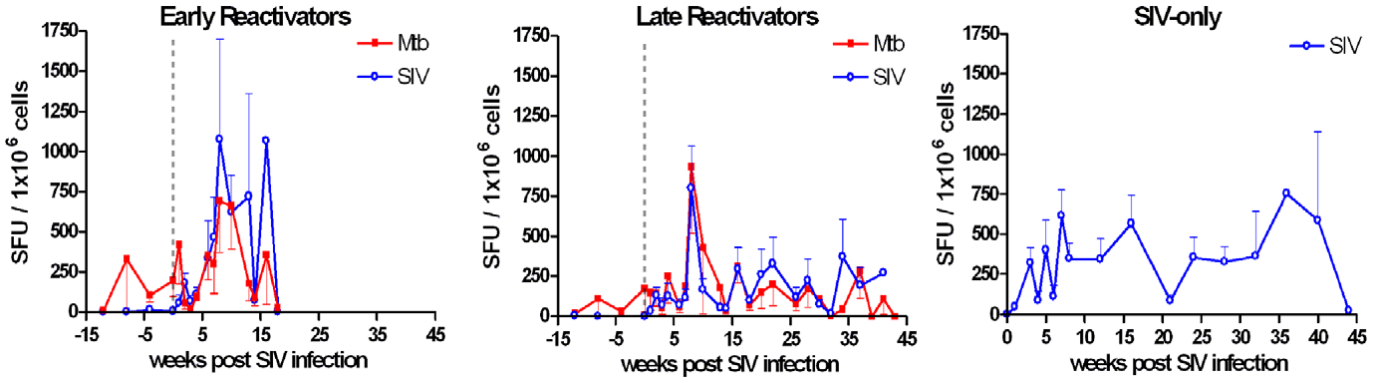
Co-infected monkeys experience an increase in numbers of Mtb-specific T cells following early after SIV infection. IFNγ ELISPOTs were performed on PBMCs stimulated with Mtb or SIV antigens. IFN-γ was measured in spot forming units (SFU) per 1×10^6^ cells.

Previous studies using IFNγ ELISPOT indicate macaques with active or reactivating TB have more IFNγ-producing PBMCs than animals with latent TB [21], [24]. Given this, we performed IFNγ ELISPOT to determine whether changes in the frequency of Mtb- or SIV-specific T cells within PBMC could predict reactivation in co-infected animals (Figure 3). Unexpectedly, numbers of Mtb- and SIV-specific T cells increased sharply in all monkeys between 7–8 weeks post SIV infection. This increase in SIV- and Mtb-specific T cells quickly declined in all late reactivators and one early reactivator. The number of SIV-specific T cells within the SIV-only group did not change considerably between 8 and 32 weeks post SIV infection. The increase in Mtb-specific T cells coincided with an increase in the expression of late (CD29) but not early (CD69) activation markers on both CD4 and CD8 T cells (Figure S3). We hypothesized that the initial burst of Mtb-specific IFNγ responses shortly after SIV infection may reflect a transient perturbation of the local control of infection at the level of the granuloma, resulting in an increase in antigen load due to bacterial replication.

### Co-Infected Monkeys Had More Pathology, Higher Bacterial Numbers, and More Dissemination than Latent Monkeys

**Figure 4:**
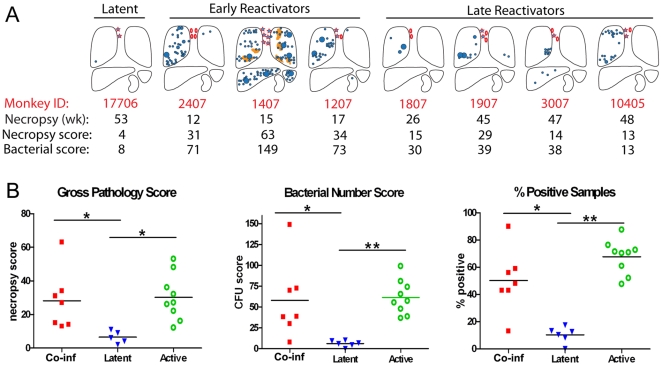
Pathology associated with reactivation in co-infected monkeys. **A.** Gross pathology at time of necropsy in co-infected monkeys. Weeks indicates time post-SIV infection for co-infected monkeys or post-Mtb infection for monkey 17706. Necropsy score represents the gross pathology score, CFU score indicates the bacterial number score. Lungs, thoracic lymph nodes, spleen, liver and kidney are represented. Granulomas are represented by blue circles, TB pneumonia by orange patches, enlarged thoracic lymph nodes by open ovals, and granulomatous thoracic lymph nodes by stars. Monkey 17706 is representative of pathology found in latent monkeys. **B.** Reactivation was quantified by gross pathology score, bacterial number score and percentage of tissue samples positive for Mtb in homogenized tissue. Co-infected monkeys are statistically different than latent monkeys (Mann-Whitney, gross pathology P = 0.0025, bacterial number score P = 0.004, % positive samples P = 0.005). Latent monkeys are also statistically different than active monkeys in gross pathology (Mann-Whitney, P = 0.001, bacterial number score P = 0.0004, and % positive P = 0.0004).

Each co-infected monkey displayed clinical indicators of TB following SIV infection that are not seen in monkeys with latent TB. Reactivated disease was confirmed at necropsy by pathology- and microbiology-based measurements that characterize the extent of visible and microscopic disease. Gross pathology at necropsy was quantified by a metric referred to as the necropsy score [18] that reflects the grossly visible tuberculous disease including the number and size of visible granulomas in each lung lobe and thoracic lymph node and extent of dissemination beyond the thoracic cavity. The monkeys that reactivated early post-SIV infection (<17 weeks) had more TB-related pathology than monkeys that reactivated later post-SIV infection, and thus had a trend towards higher necropsy scores (P = 0.0571, Figure 4A). We also scored the total bacterial burden for each monkey, and the percent of tissues sampled at necropsy that were Mtb culture-positive. A bacterial number score [18] was used to reflect the total bacterial load and was obtained by summing the log-transformed number of colony forming units (CFU) from all sampled tissues. The percent positive score measured the proportion of sampled tissues that are Mtb culture positive, thereby indicating the extent of bacterial dissemination in visibly involved and uninvolved tissues. The co-infected monkeys had gross pathology, percent positive and bacterial number scores that were similar to SIV-negative monkeys with active TB and significantly higher than what is seen in latent monkeys (Figure 4B). These metrics, therefore, support the pre-necropsy clinical data indicating that the co-infected monkeys had disease consistent with reactivated TB.

### Histology Reveals a Mixture of Chronic and Active Granulomas in Co-Infected Monkeys

The granulomas seen in active and latent monkeys have been described previously and are extremely similar to those seen in human TB [18], [21], [29]. To summarize, monkeys classified as having latent infection typically have at least one granuloma in one lung lobe and associated draining thoracic lymph node(s). These granulomas are generally caseous with partial or total mineralization, often combined with extensive peripheral fibrous connective tissue deposition [18]. Completely fibrotic (sclerotic) lesions are occasionally observed. In contrast, monkeys with active TB frequently have a range of lesion types, including caseous with or without peripheral fibrosis, non-necrotizing (primarily epithelioid macrophages with a lymphocytic component), and suppurative (with significant neutrophilic infiltrate) [18]. Mineralized or completely fibrotic lesions indicative of more chronic immunologic responses to subclinical (latent) infection are rarely observed in monkeys with active TB.

**Figure 5:**
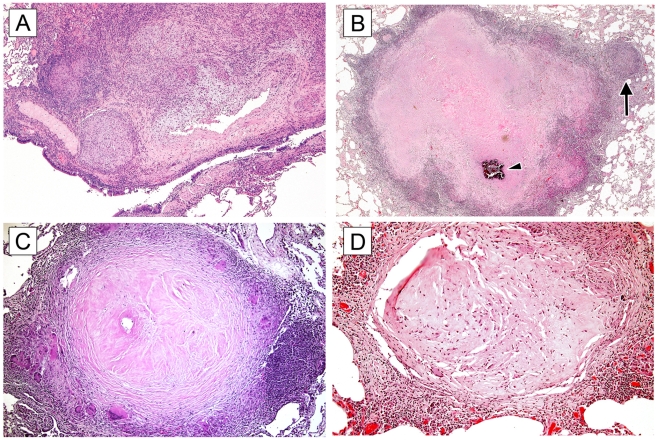
Histopathology associated with co-infected monkeys. **A.** Actively disseminating tuberculous disease characterized by granulomatous inflammation infiltrating into the wall of a large bronchus and adjacent vessel. Hematoxylin and eosin (H&E) stain, 5× magnification. **B.** A “satellite” nodule (arrow) comprised of non-necrotizing granulomatous inflammation extends from the outer margin of a more chronic caseous granuloma, suggesting loss of immunological containment and reactivation. Arrowhead indicates mineralized area. H&E stain, 2× magnification C. A chronic granuloma with extensive central fibrous organization in conjunction with a dense circumscribing margin of peripheral fibrosis. H&E stain, 10× magnification. D. A solid fibrotic granuloma comprised of maturing fibroblasts depositing a collagenous matrix. H&E stain, 10× magnification.

In general, the co-infected monkeys had a mix of active and chronic lesions (Table 2). Monkey 1407, which reactivated early post-SIV infection, had extensive widespread active lesions with little evidence of chronic disease. Monkeys 2407 and 1207, which also reactivated early post SIV infection, had pathology consistent with disseminated active TB (Figure 5A), but additionally showed some chronic appearing lesions, including a few solid fibrotic lesions. Certain chronic caseous granulomas had signs of reactivation foci around the perimeter (Figure 5B) as indicated by the presence of macrophage and lymphocyte-rich “satellite” granulomas associated with reactivation [18], [24]. Monkeys that reactivated later post-SIV infection experienced a range of involvement, with 3007 and 1907 having more active-type lesions than 10405 and 1807, although these monkeys as a group had fewer active-appearing lesions than the animals that reactivated earlier following SIV-infection. Additionally, there were more mineralized granulomas reflecting the original latent infection. However, a striking finding was the presence of substantially more completely fibrotic granulomas, especially in 3007, (Figure 5C, 5D and Table 2) than is normally noted in active or latent monkeys. In our experience to date, these completely fibrotic granulomas are usually seen in monkeys that had active disease but were then treated with anti-tuberculous drugs for 1–2 months (unpublished data), strongly suggesting that this phenotype represents healing of active granulomas. These granulomas are occasionally seen in latent monkeys, but are present in a higher proportion of granulomas examined histologically from the co-infected monkeys (Table 2). Table 2 also demonstrates that many more granulomas were found in the co-infected monkeys than in Mtb-only latent monkeys, which reflects reactivation and likely the spread of infection. Taken in the context of the co-infection, a possible interpretation of the increased presence of completely fibrotic granulomas is that SIV infection perturbed the local control of the latent infection, leading to subclinical reactivation in some monkeys and spread of infection in the lungs, with subsequent healing of some granulomas as the host controlled virus load and regained control of the Mtb infection.

**Table 2:** Granulomas observed in Mtb-only and SIV-Mtb co-infected monkey.

	Total number of monkeys	Granulomas examined	Caseous	Non-necrotic	Mineralized	Completely fibrotic
Active	4	224	62%	36%	1%	1%
Mtb-SIV: *early*	3	169	39%	57%	3%	1%
Mtb-SIV: *Late*	4	82	15%	52%	7%	26%
Latent	5	23	4%	0%	82%	13%

### IFNγ Production in Tissues Was Not Affected by SIV Infection

**Figure 6:**
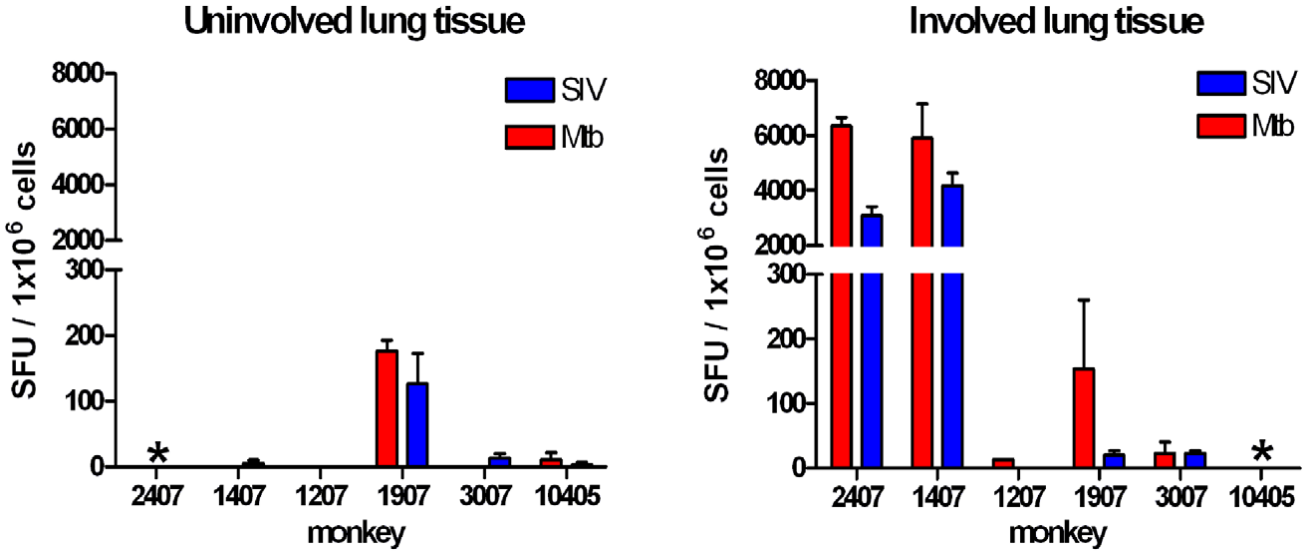
Antigen specific T cells in involved and uninvolved tissue of co-infected monkeys. Tissue homogenates were isolated from the granulomatous (involved) or tissues without grossly present granulomas (uninvolved) in lung. Monkeys not represented because too few cells were recovered from tissues at necropsy are indicated by asterisks.

IFNγ ELISPOTs were performed on tissue homogenates of lung lobes at necropsy to determine whether SIV infection impaired anti-mycobacterial IFNγ responses (Figure 6). These tissues were separated into tissue containing grossly visible granulomas (involved) and those without (uninvolved). Uninvolved lung tissue did not demonstrate a strong response to either Mtb or SIV peptides in any monkeys, except 1907 which had Mtb-culture positive tissues that did not contain visible granulomas, but likely had microscopic disease. Two monkeys had very high frequencies of Mtb and SIV-specific IFNγ-producing cells in involved lung tissue; these monkeys also had the most disease and highest bacterial burdens. Previously, we demonstrated that IFNγ producing T cells in the tissues of monkeys are higher in monkeys with more disease, likely reflecting the amount of antigen present in the tissues, due to higher numbers of bacilli [18]. IFNγ responses to SIV or Mtb antigens in uninfected and infected thoracic lymph node cells were not significantly different (data not shown). Co-infected monkeys had similar frequencies of SIV-specific IFNγ-releasing cells compared to SIV-only monkeys (data not shown).

### Correlation between Mycobacterial CFU and Viral Titer

It has been reported that TB-involved lung lobes have higher virus loads than uninvolved lobes in humans [30], suggesting that Mtb-related inflammation infection can increase viral replication. To address this, we directly sampled granulomas and other tissues at necropsy to measure bacteria and virus loads. While we found a broad range of Mtb and SIV loads, both between monkeys and within the same monkey, there was no correlation of viral titers with bacterial loads in either granulomas or thoracic lymph nodes (Table S1).

### Co-Infected Monkeys Had Fewer T Cells in Involved Tissues than SIV- or Mtb-Only Animals

To determine that SIVmac251 depleted CD4 T cells in mucosal (uninvolved) tissue, we compared CD4 and CD8 T cell frequencies in gut tissue in monkeys with infected with Mtb (no SIV), co-infected monkeys and an SIV-only monkey. There was substantial depletion of CD4 T cells in the colon (25.3% Mtb, 5.7% co-infected) and ileum/jejunum (14.0% Mtb, 1.6% co-infected, 8.6% SIV only). CD8 T cells were only slightly decreased in the co-infected monkey gut tissues compared to controls. These data indicate that SIVmac251 is capable of reducing T cells in the mucosal tissue, as has been previously reported for rhesus macaques [31] and for humans (with HIV) [32].

**Figure 7:**
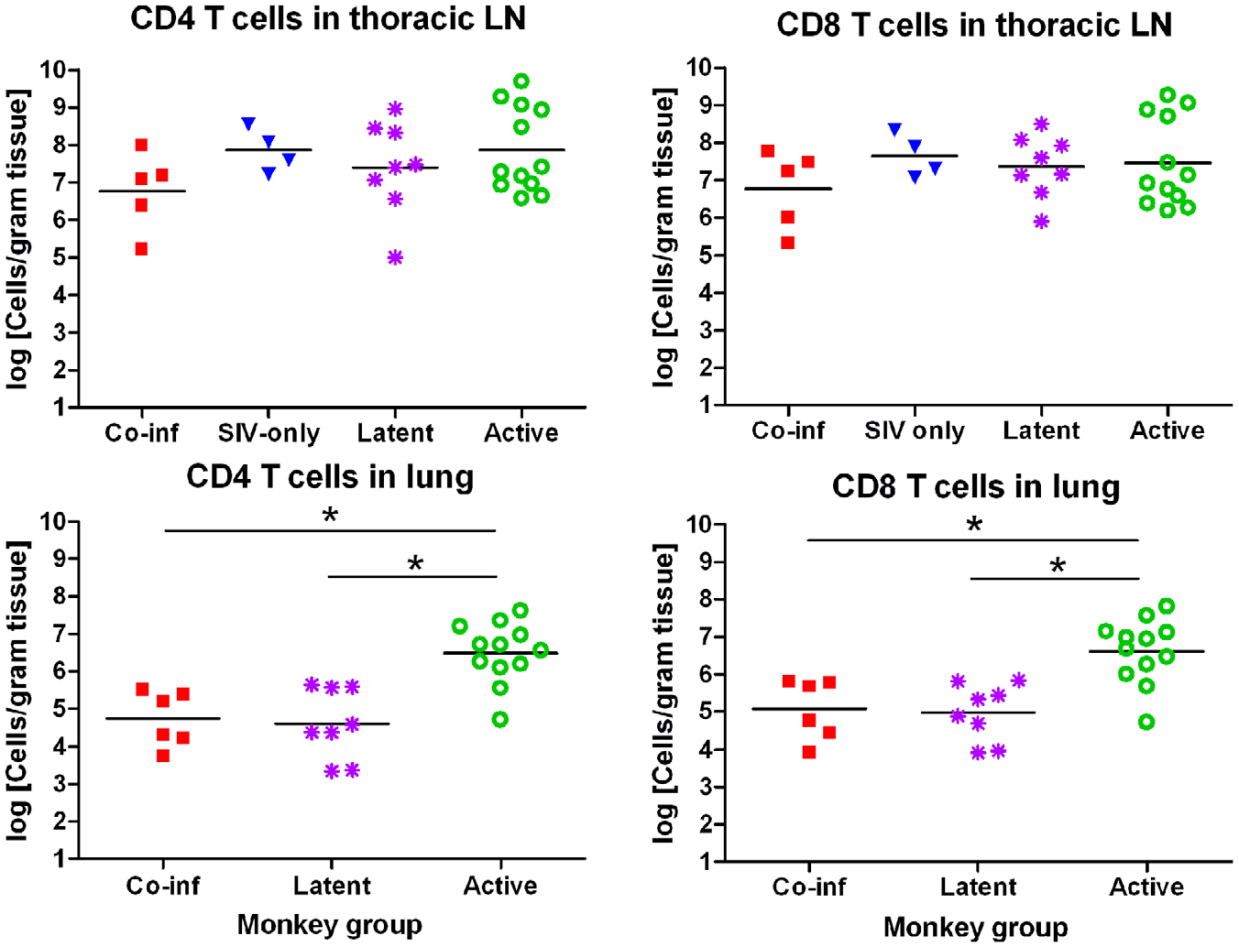
CD4 and CD8 T cell numbers in lung and thoracic lymph nodes. CD4 and CD8 T cell numbers in lung-draining thoracic lymph nodes (LN) are not significantly different between co-infected animals and animals with latent or active TB. T cell numbers in co-infected lung tissues were similar to animals with latent TB but significantly lower than animals with active TB (Mann-Whitney, CD4 P = 0.0023; CD8 P = 0.0078). Latent monkeys have fewer lung T cells than active monkeys (Mann-Whitney, CD4 P = 0.0014; CD8 P = 0.0018). Data points represent average T cell numbers from either thoracic LN or lung tissue from each monkey. Not all co-infected monkeys are not present due to limited tissue availability.

We measured CD4 and CD8 T cell numbers in draining lymph nodes and TB-involved lung from co-infected monkeys to better understand how SIV infection affects cell numbers in tissues infected with TB. A trend of fewer CD4 T cell numbers in the thoracic lymph nodes of co-infected monkeys compared to SIV-only monkeys was observed (Figure 7). Viral titers in the draining lymph nodes of co-infected monkeys and SIV-only monkeys were not significantly different (data not shown). T cell numbers in lung tissue were also examined although we did not obtain sufficient numbers of T cells from lung tissue from SIV-only monkeys for comparison, probably because these monkeys did not have pulmonary infections with other pathogens. As previously reported, T cell numbers are higher in involved lung tissue from active monkeys compared to latent monkeys [18]. Here, the co-infected monkeys had T cell numbers similar to the number observed in latent monkeys (Figure 7) even though they had significant disease and increased bacterial numbers, similar to active TB monkeys. This observation suggests co-infected monkeys may have critical defects in their ability to mount or maintain T cell-mediated immune responses at the site of infection.

## Discussion

Reactivated TB is a major source of morbidity and mortality among HIV-infected individuals. While most opportunistic infections commonly occur when CD4 T cell numbers have significantly declined, TB occurs throughout the entire spectrum of HIV disease, including when CD4 T cells numbers are well preserved and stable [4], [5], [6]. Despite the severe risk of TB in HIV-infected persons, research into factors underlying early reactivation has been hampered by lack of appropriate animal models combining latent TB and HIV infection. In this study, cynomolgus macaques with latent TB were infected with SIVmac251 to investigate the factors that could lead to reactivation of latent TB. All the monkeys with latent TB co-infected with SIV experienced clinical signs, pathology and bacterial load consistent with reactivated disease while the Mtb-negative SIV-infected monkeys did not develop simian AIDS or opportunistic infections within 11 months of infection. SIV-negative cynomolgus macaques with latent TB have a <5% chance of undergoing reactivation and can maintain latent TB without reactivation for more than three years (unpublished data). These observations suggest that while these monkeys can control either SIV or latent Mtb infection independently, SIV-induced immune dysfunction disrupted the immunologic pressure constraining mycobacterial growth, inducing reactivation. To our knowledge, this is the first animal model of latent TB where the immunologic and microbiologic responses to SIV co-infection can be directly investigated.

Although individuals are at a high risk of developing TB within the first year of HIV-seroconversion [33], clinical studies of HIV-Mtb co-infection indicate that depletion of peripheral CD4 T cell numbers is not predictive of when individuals are most likely to experience TB [6]. However HIV+ individuals with low peripheral CD4 T cells have been shown to be at greater risk of developing active TB [34]. All the monkeys experienced T cell depletion during acute SIV infection, with reactivated TB occurring during the period when the animals had regained normal and stable peripheral T cell numbers. Our data suggests that rather than the T cell numbers during chronic SIV infection being a major factor in reactivation, the extent of initial depletion and post-acute recovery may affect ultimate control of latent Mtb infection. Monkeys 1407 and 2407, which reactivated early post-SIV, had fewer peripheral T cells than other monkeys and didn't recover from the initial depletion as well as monkeys that reactivated later. Similarly, 1807 and 1907, which also had fewer peripheral T cells following SIV infection, displayed signs of active disease earlier than the other late reactivators, supporting the hypothesis that early perturbations of the immune response may ultimately affect disease progression. In contrast with this, we also infected two latent cynomolgus macaques with SHIV89.6 (data not shown). These monkeys had significant sustained CD4 T cell depletion in the periphery as has been reported for cynomolgus macaques infected with SHIV89.6 [27], yet did not experience reactivation by seven months post-SHIV infection (unpublished data). Although SHIV89.6 induces a dramatic loss of peripheral CD4 T cells it did not cause significant decreases of mucosal CD4 T cells (unpublished data). Taken together, these data support the conventional paradigm that peripheral CD4 T cell populations are imperfect indicators of an individual's susceptibility to TB, and instead indicate that early T cell depletion by SIV (or perhaps HIV) that depletes both peripheral and mucosal CD4 T cells may predict risk of reactivation.

Although the link between peripheral cells and maintenance of latency is not clear, depletion in the periphery shortly after SIV infection may reflect significant depletion of T cells in the granuloma. Monkeys 1407 and 2407, which didn't recover well from acute SIV infection and reactivated early, displayed more disease than later reactivators, possibly indicating that significant disruption occurred within granulomas and the subsequent inability to replace T cells lead to reactivated TB. This link between events in the periphery and the local granuloma environment is supported by the decreases observed in the frequency of CD4 T cells in the airways from the early-reactivating macaques. The acute phase of SIV infection was accompanied by increased numbers of Mtb-specific IFNγ-producing T cells in the periphery. This may be caused by SIV-induced impairment of granuloma function leading to increased mycobacterial growth and dissemination, which results in increased amounts of bacterial antigen released. Taken together, these data suggest that systemic T cell depletion permits resumption of bacterial growth and perhaps reactivation. Indeed, HIV-induced depletion of Mtb-specific CD4 T cells in the granuloma, and subsequent disruption of cellular architecture critical [27] for bacterial containment, have been hypothesized as factors contributing to increased reactivation rates in co-infected humans [9], [35].

The extensive histological examination of granuloma-containing tissue from animals with widely different amounts of disease expanded our basic understanding of how HIV may change the pathology of tuberculosis. Granulomas from the earliest-reactivating animals were most similar to animals with active disease [18] whereas macaques that reactivated later had more mineralized granulomas, which are most often associated with latent disease, with evidence for some localized dissemination and reactivation. The lungs of latent monkeys normally contain few granulomas, and they are usually either completely calcified lesions or caseous ones with significant mineralization, with an occasional completely fibrotic granuloma in some monkeys. The most striking finding in the co-infected monkeys was the presence of numerous completely fibrotic granulomas in the lungs, which are rarely seen in latent infection [18]. We have observed this granuloma type in the setting of short-term drug treatment in the macaque model (unpublished data), and thus associate this fibrosis with a healing response. Our interpretation of this unusual pathology is that the initial events surrounding SIV infection caused a depression in the local immune response (the granulomas) in latent animals, allowing increased bacterial growth, spread to other areas of the lung, and formation of additional granulomas. After this initial event, some animals may regain partial control of the infection, and these new granulomas begin to heal and become fibrotic. Thus, the interaction of SIV with the latent TB granuloma is a dynamic, and can lead to various outcomes. The cytokine milieu in the SIV-infected lungs may also promote more fibrotic-type healing, leading to the abundance of this type of lesion; this hypothesis requires further study.

There was a trend of fewer CD4 T cells in the pulmonary lymph nodes of co-infected monkeys compared to SIV-only monkeys, indicating that the presence of mycobacteria, and perhaps the increased T cell activity in the lymph nodes, leads to more depletion of these cells in the lymphoid tissue, and may contribute to enhanced disease. Co-infected monkeys had fewer CD4 and CD8 T cells within TB-involved lung tissues when compared to SIV-negative monkeys with active TB. This contradicts what normally happens in animals with active TB, which have more T cells in involved tissues than latent monkeys. Significant reductions in T cell numbers within the lung tissue of the co-infected monkeys suggest that SIV is interfering with migration of T cells to the site of infection as has been seen with HIV [36]. Alternatively, SIV may be inducing T cell death within the granuloma like that observed in HIV-Mtb co-infected humans [37]. Exposure to Mtb antigens is linked to apoptosis in HIV-infected cells [38], which could mechanistically explain why tissues in co-infected animals were depleted when compared with animals only infected with Mtb. To our knowledge, this study provides the first evidence demonstrating quantitative T cell depletion within granulomatous tissue, supporting hypotheses for mechanisms by which HIV disrupts the maintenance of latent Mtb infection [8], [9].

Several groups have investigated the effects of HIV-Mtb co-infection on the replication of both pathogens [39], [40], [41], [42] and the relationship between virus load and disease progression [7], [43], [44]. We found similar peripheral and tissue virus loads in co-infected and SIV-only monkeys, suggesting that virus loads were not influenced by Mtb infection. Granulomatous tissue might be an ideal environment for HIV replication as abundant T cells, increased cell-cell interaction, and high TNF expression promote cell-cell transmission and virus replication [9], [40], [42]. We were unable to confirm this, however, and could not correlate virus loads with Mtb burden in individual granulomas (Table S1). Differences in viral titer did not appear to be the cause of reactivation either, as viral titers (peak and during the chronic phase) were similar between the early and late reactivators. Similar findings have come from the HIV-TB clinical literature [45], [46], highlighting the clinical relevance of the cynomolgus macaque co-infection model. Our viral titers were relatively low following the acute infection, however, and it is possible that higher viral titers in a different macaque species or with a different virus strain could lead to different results. Even with the low virus loads observed in these cynomolgus macaques they all experienced reactivation of latent TB.

We have developed a tractable animal model of reactivated TB in HIV-co-infected humans. Although numerous clinical studies have been undertaken to investigate HIV-Mtb co-infection, most examine patients with active TB and many are either retrospective or limited by the number and types of tissues and time points that could be sampled. This novel model will be a useful tool for investigating interactions between Mtb and HIV, and the immunologic events that lead to reactivation of latent TB or exacerbation of primary infection.

## Supporting Information

Figure S1 Schematic representation of experimental design. Two groups of animals were established, a SIV-only control group and a Mtb-SIV co-infection group. Historical controls based on other studies were used as Mtb-only controls for active or latent TB. Green indicates the time where Mtb-only, pre-SIV infection baseline data were acquired. Red indicates 0–8 weeks post-SIV infection where blood was drawn weekly, BAL cells and gastric aspirates were acquired every four weeks and lymph node biopsies were done at four and eight weeks post-SIV. Light blue (SIV-only) indicates where monthly blood draws and BAL procedures were performed. Dark blue (Mtb-SIV co-infected) indicates where blood draws for ELISPOT assays and virus load determination were performed every other week, BAL cells and gastric aspirates were acquired every four weeks and lymph node biopsies were performed at weeks 12 and 24 post-SIV infection. No SIV-only animals experienced any sAIDS-like symptoms.
(0.38 MB TIF)

Figure S2 Changes in T cell frequencies in the peripheral lymph nodes of co-infected macaques. Peripheral (inguinal or axillary) lymph node biopsies were performed on co-infected monkeys at 0, 4, 12, and 24 weeks post SIV inoculation. Numbers represent early (1207, 1407, 2407) and late (1807, 1907, 3007, 10405) reactivator.
(5.40 MB TIF)

Figure S3 T cell activation markers increase following SIV infection. Changes in the expression of early (CD69) and late (CD29) activation markers are represented in CD4 or CD8 peripheral T cells over time.
(8.63 MB TIF)

Table S1 Virus and bacterial burden in uninvolved and involved tissues.
(0.05 MB DOC)
