# Correction: Reactivation of Latent Tuberculosis in Cynomolgus Macaques Infected with SIV Is Associated with Early Peripheral T Cell Depletion and Not Virus Load

**DOI:** 10.1371/journal.pone.0124221

**Published:** 2015-04-13

**Authors:** The PLOS ONE Staff

The authors incorrectly report that 10^6^–10^7^ TCID50 units of SIVmac251 were intravenously injected into the macaques. The amount of virus actually injected was approximately 1.67x10^5^ RNA copies. This does not affect the data or conclusions.
