# Supplementary figures and images for: Reactivation of Latent Tuberculosis in Cynomolgus Macaques Infected with SIV Is Associated with Early Peripheral T Cell Depletion and Not Virus Load
Source: PLoS One. 2010 Mar 10;5(3):e9611. doi: 10.1371/journal.pone.0009611 (PMC2835744; doi:10.1371/journal.pone.0009611)

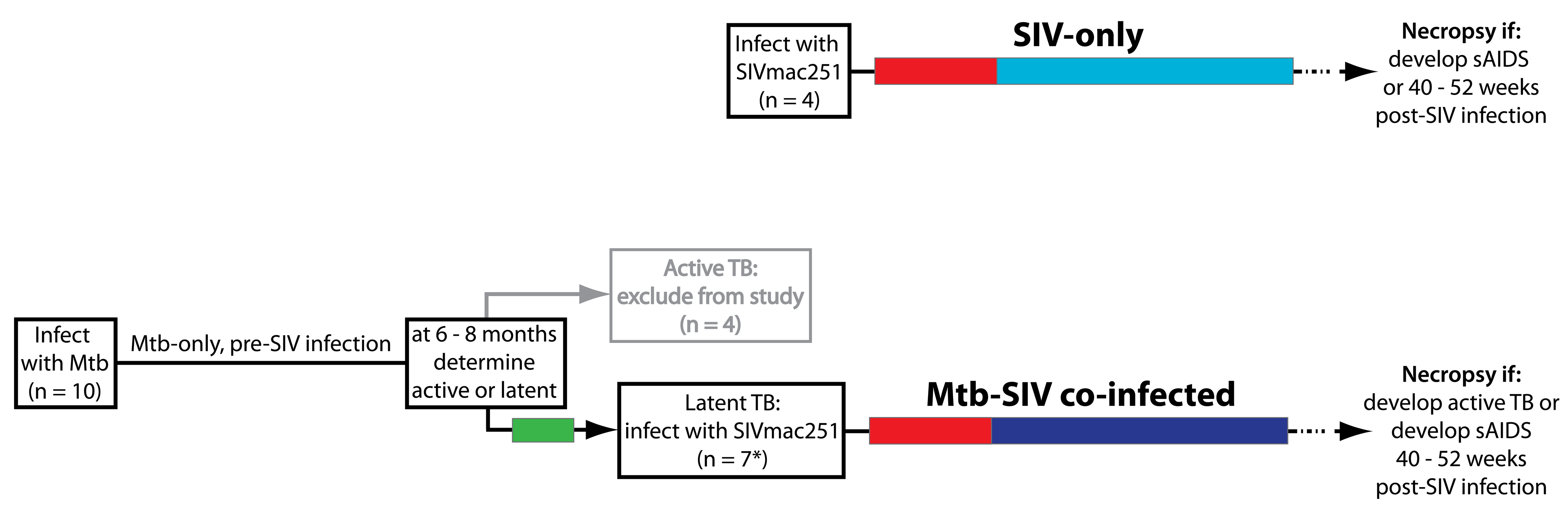

Supplement: Figure S1 — Schematic representation of experimental design. Two groups of animals were established, a SIV-only control group and a Mtb-SIV co-infection group. Historical controls based on other studies were used as Mtb-only controls for active or latent TB. Green indicates the time where Mtb-only, pre-SIV infection baseline data were acquired. Red indicates 0–8 weeks post-SIV infection where blood was drawn weekly, BAL cells and gastric aspirates were acquired every four weeks and lymph node biopsies were done at four and eight weeks post-SIV. Light blue (SIV-only) indicates where monthly blood draws and BAL procedures were performed. Dark blue (Mtb-SIV co-infected) indicates where blood draws for ELISPOT assays and virus load determination were performed every other week, BAL cells and gastric aspirates were acquired every four weeks and lymph node biopsies were performed at weeks 12 and 24 post-SIV infection. No SIV-only animals experienced any sAIDS-like symptoms. (0.38 MB TIF) [file pone.0009611.s001.tif]

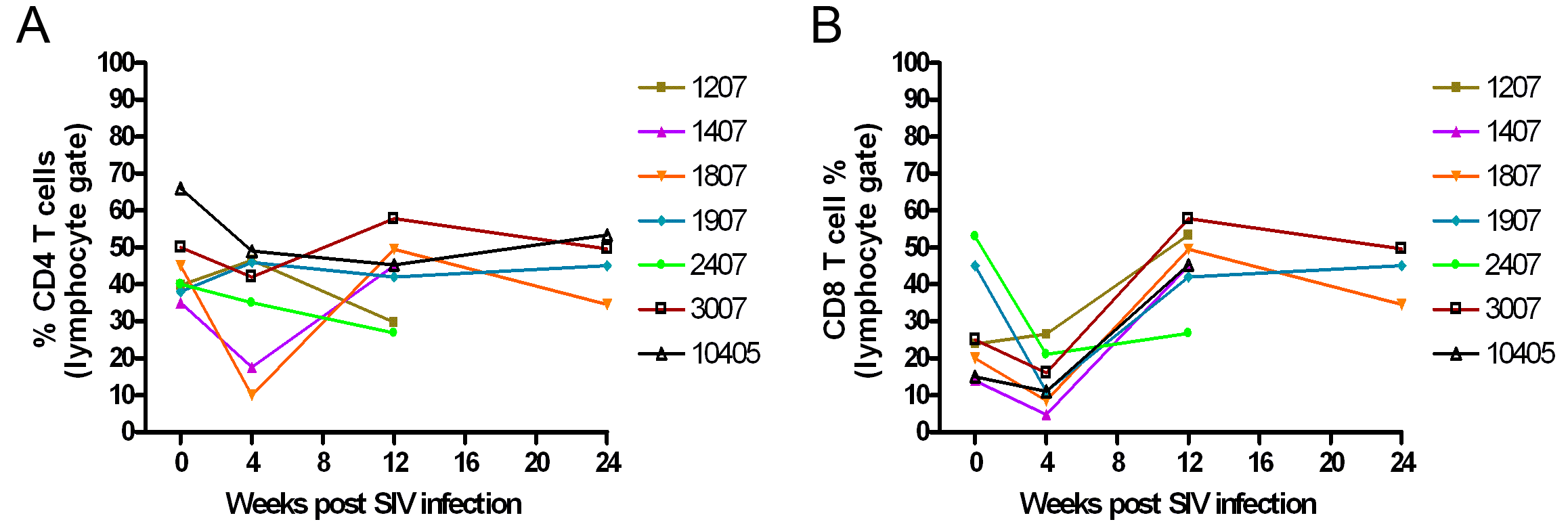

Supplement: Figure S2 — Changes in T cell frequencies in the peripheral lymph nodes of co-infected macaques. Peripheral (inguinal or axillary) lymph node biopsies were performed on co-infected monkeys at 0, 4, 12, and 24 weeks post SIV inoculation. Numbers represent early (1207, 1407, 2407) and late (1807, 1907, 3007, 10405) reactivator. (5.40 MB TIF) [file pone.0009611.s002.tif]

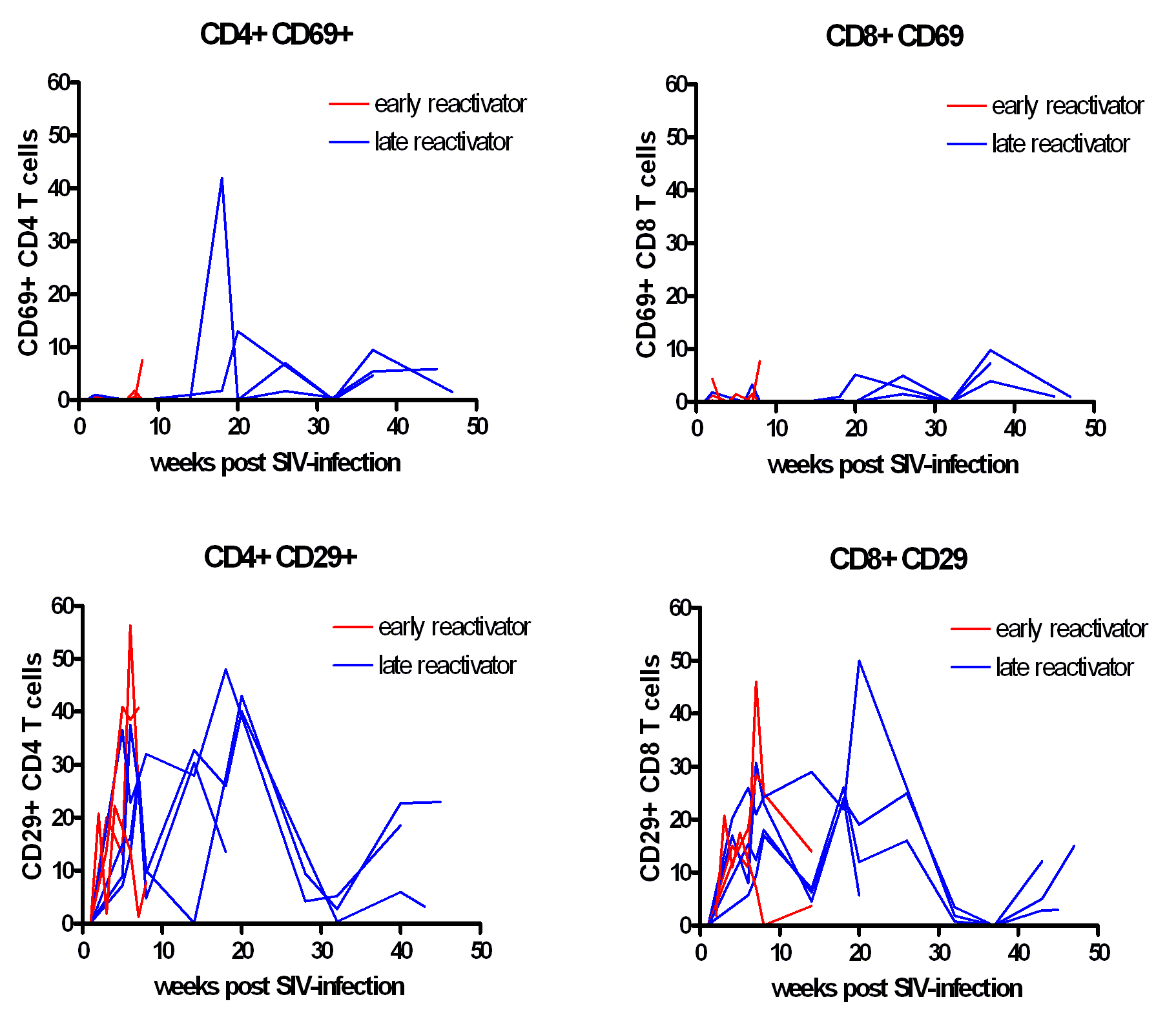

Supplement: Figure S3 — T cell activation markers increase following SIV infection. Changes in the expression of early (CD69) and late (CD29) activation markers are represented in CD4 or CD8 peripheral T cells over time. (8.63 MB TIF) [file pone.0009611.s003.tif]
