# Supplementary material for: Reactivation of Latent Tuberculosis in Cynomolgus Macaques Infected with SIV Is Associated with Early Peripheral T Cell Depletion and Not Virus Load
Source: PLoS One. 2010 Mar 10;5(3):e9611. doi: 10.1371/journal.pone.0009611 (PMC2835744; doi:10.1371/journal.pone.0009611)
Supplement: Table S1 — Virus and bacterial burden in uninvolved and involved tissues. (0.05 MB DOC) [file pone.0009611.s004.doc]

**Table S1.** *Virus and bacterial burden in uninvolved and involved tissues.*

| **Tissue** |  | **Early Reactivators** | | |  | **Late Reactivators** | | | |
| --- | --- | --- | --- | --- | --- | --- | --- | --- | --- |
| **Uninvolved lung** |  | **1207** | **1407** | **2407** |  | **1807** | **1907** | **3007** | **10405** |
| CFU/gram tissue |  | 4.0x10^2^ | 1.3x10^2^ | 1.4x10^5^ |  | 1.2x10^2^ | 2.5x10^3^ | 4.2x10^2^ | 7.4x10^2^ |
| SIV titer (copies/10^6^ cells) |  | 9.1x10^2^ | <50 | 1.2x10^4^ |  | 1.2x10^3^ | 5.0x10^4^ | 3.0x10^3^ | <50 |
|  |  |  |  |  |  |  |  |  |  |
| **Involved lung** |  | **1207** | **1407** | **2407** |  | **1807** | **1907** | **3007** | **10405** |
| CFU/gram tissue |  | 2.0x10^4^ | 3.7x10^4^ | 2.5x10^3^ |  | 6.3x10^3^ | 7.4x10^3^ | 4.4x10^4^ | 6.0x10^2^ |
| SIV titer (copies/10^6^ cells) |  | 1.3x10^4^ | <50 | <50 |  | 6.5x10^3^ | 3.0x10^5^ | 2.7x10^3^ | <50 |
|  |  |  |  |  |  |  |  |  |  |
| **Uninvolved lymph node^a^** |  | **1207** | **1407** | **2407** |  | **1807** | **1907** | **3007** | **10405** |
| CFU/gram tissue |  | 7.3x10^2^ | 8.7x10^4^ | 1.9x10^3^ |  | 5.8x10^2^ | 0 | 4.4x10^3^ | 0 |
| SIV titer (copies/10^6^ cells) |  | 1.7x10^3^ | 3.7x10^2^ | 2.0x10^4^ |  | 1.4x10^5^ | 3.7x10^5^ | 5.4x10^4^ | <50 |
|  |  |  |  |  |  |  |  |  |  |
| **Involved lymph node^a^** |  | **1207** | **1407** | **2407** |  | **1807** | **1907** | **3007** | **10405** |
| CFU/gram tissue |  | 7.8x10^3^ | 1.4x10^5^ | NA^b^ |  | 1.3x10^4^ | 1.6x10^3^ | 4.0x10^4^ | 0 |
| SIV titer (copies/10^6^ cells) |  | 6.4x10^2^ | 1.7x10^2^ | NA^b^ |  | 4.0x10^5^ | 5.8x10^5^ | 1.2x10^4^ | 6.0x10^1^ |

^a^ Lymph nodes are lung-draining thoracic lymph nodes

^b^ NA indicates tissues not available for analysis
